# Interaction Analysis of *VDR* rs7975232 Polymorphism and the Triglyceride-Glucose Index in Relation to Metabolic Outcomes in Older Adults

**DOI:** 10.3390/ijms27209252

**Published:** 2026-10-18

**Authors:** Szymon Michniewicz, Krzysztof Chmielowiec, Jolanta Chmielowiec, Bartłomiej Czyżniewski, Mathias Z. Strowski, Ewa Pruszyńska-Oszmałek, Paweł Kołodziejski, Michał T. Kowalski, Anna Grzywacz, Magdalena Gibas-Dorna

**Affiliations:** 1 Institute of Health Sciences, Collegium Medicum, University of Zielona Góra, Zyty Str. 28, 65-046 Zielona Góra, Poland; s.michniewicz@inz.uz.zgora.pl (S.M.); j.chmielowiec@inz.uz.zgora.pl (J.C.); 2 Institute of Medical Sciences, Collegium Medicum, University of Zielona Gora, 28 Zyty Street, 65-046 Zielona Góra, Poland; b.czyzniewski@inz.uz.zgora.pl (B.C.); m.gibas-dorna@inz.uz.zgora.pl (M.G.-D.); 3 Department of Hepatology and Gastroenterology, Interdisciplinary Centre of Metabolism: Endocrinology, Diabetes and Metabolism, Charité-University Medicine Berlin, 13353 Berlin, Germany; mathias.strowski@charite.de; 4 Department of Animal Physiology, Biochemistry and Biostructure, Poznan University of Life Sciences, 35 Wołynska Street, 60-637 Poznan, Poland; ewa.pruszynska@up.poznan.pl (E.P.-O.); pawel.kolodziejski@up.poznan.pl (P.K.); 5 Clinical Department of Cardiology, Multispecialty Hospital in Nowa Sol, 7 Chalubinskiego Street, 67-100 Nowa Sol, Poland; kowaltmd@wp.pl; 6 Department of Public Health, Epidemiology, and Behavioral Genetics, Pomeranian Medical University, 70-111 Szczecin, Poland; anna.grzywacz@pum.edu.pl

**Keywords:** vitamin D receptor (VDR), *VDR* rs7975232, triglyceride–glucose (TyG) index, non-esterified fatty acids (NEFA), atherogenic index of plasma (AIP), insulin resistance, glucose–lipid metabolism, older adults

## Abstract

Ageing is associated with increasing metabolic heterogeneity driven by interactions between adipose tissue dysfunction and altered glucose-lipid metabolism. The vitamin D receptor (VDR) rs7975232 polymorphism has been investigated in relation to metabolic traits; however, its relationship with glucose–lipid metabolic characteristics remains unclear. This study aimed to investigate the association of the *VDR* rs7975232 polymorphism with metabolic markers and whether their relationships with the TyG index differ according to genotype in older adults. The study included 95 older adults living in a long-term care facility (mean age 62.71 ± 14.13 years). Anthropometric and fasting biochemical parameters were assessed, and the triglyceride–glucose (TyG) index, homeostatic model assessment of insulin resistance (HOMA-IR), and atherogenic index of plasma (AIP) were calculated. *VDR* rs7975232 genotyping was performed using real-time PCR. Multivariable linear regression models assessed genotype × TyG interactions after adjustment for age, sex, and waist-to-hip ratio. The rs7975232 genotype was not independently associated with TyG, NEFA, AIP, or other metabolic characteristics. In multivariable models using continuous TyG, genotype × TyG interactions were not statistically significant for NEFA or AIP. A nominal interaction was observed for fasting glucose, whereas no significant interactions were observed for log-transformed fasting insulin or log-transformed HOMA-IR. None of the genotype × TyG interactions remained statistically significant after Benjamini–Hochberg false-discovery-rate correction. In the exploratory categorical sensitivity analysis, AA genotype × higher TyG category interactions had nominal *p*-values < 0.05 for NEFA and AIP. In this exploratory cross-sectional cohort, *VDR* rs7975232 was not independently associated with the assessed metabolic traits. The primary analyses did not provide robust evidence of genotype-dependent differences in TyG–metabolic associations. Nevertheless, exploratory analysis using categorized TyG suggested potential genotype-dependent differences in associations with NEFA and AIP. These findings are hypothesis-generating and require confirmation in larger independent cohorts.

## 1. Introduction

Ageing is associated with profound alterations in adipose tissue distribution and metabolic function, including increased visceral adiposity, ectopic lipid accumulation, impaired adipogenesis, and chronic low-grade inflammation. Together, these changes contribute to insulin resistance, dyslipidemia, and increased metabolic vulnerability through complex disturbances in glucose and lipid metabolism [1,2,3]. Such age-related alterations may be particularly relevant in older adults living in long-term care settings, where multimorbidity, reduced mobility, and age-related physiological changes may contribute to metabolic heterogeneity [4,5]. Despite relatively standardized living conditions in long-term care facilities, substantial inter-individual variability in metabolic phenotypes persists, suggesting that factors beyond environmental exposure may contribute to this variability. This possibility is supported by longitudinal metabolomics studies demonstrating that many metabolic traits remain stable over time and exhibit significant heritability, indicating that metabolic individuality may partly reflect persistent biological factors [6].

Importantly, the biological mechanisms underlying this metabolic heterogeneity may not be fully reflected by single metabolic parameters, as ageing-related alterations in glucose regulation, lipid metabolism, and adipose tissue function may occur simultaneously and interact with each other. Therefore, identifying biological factors associated with inter-individual differences in metabolic profiles represents an important aspect of understanding cardiometabolic risk in older adults.

Among the molecular pathways implicated in adipose tissue dysfunction and metabolic ageing, vitamin D signaling has emerged as an important modulator of adipocyte biology, lipid metabolism, insulin sensitivity, and inflammatory response [7,8]. Rather than acting solely through circulating vitamin D concentrations, its biological effects depend on tissue-specific responsiveness mediated by the vitamin D receptor (VDR), a nuclear transcription factor expressed in adipose tissue, skeletal muscle, liver, pancreatic β-cells, and immune cells [9].

VDR-mediated signaling in adipose tissue plays an important role in maintaining metabolic homeostasis by regulating adipocyte differentiation, lipid storage, fatty acid metabolism, and inflammatory responses [10,11]. Consequently, genetic variability in the *VDR* gene may be associated with inter-individual variation in metabolic phenotypes [8,12].

Age-related remodeling of adipose tissue, including alterations in lipid handling, endocrine activity, and inflammatory regulation, may further increase metabolic heterogeneity among older adults [2,3].

Several common *VDR* polymorphisms have therefore been investigated in relation to metabolic traits, particularly those associated with obesity, insulin resistance, dyslipidemia, and metabolic syndrome; however, their associations with metabolic traits remain inconsistent across populations and clinical settings [12,13]. The observed heterogeneity across studies may reflect differences in age, metabolic status, environmental exposures, and gene-environment interactions. Importantly, these inconsistent findings suggest that associations between *VDR* variants and metabolic traits may vary according to biological and clinical context.

Among the commonly studied *VDR* variants, rs7975232 (ApaI), located in the 3′ untranslated region (3′UTR) of the *VDR* gene, has been investigated in relation to obesity, insulin resistance, metabolic syndrome, and other metabolic disorders, although the underlying biological mechanisms remain incompletely understood [14,15]. Previous studies have investigated rs7975232 in relation to insulin resistance-related conditions and metabolic syndrome, with some studies also reporting associations with lipid-related traits, providing the rationale for selecting this variant for the present analysis [16,17,18,19]. However, the functional significance of rs7975232 remains uncertain, and reported associations may also reflect linkage disequilibrium with other functional *VDR* variants rather than a direct biological effect of rs7975232.

Although fasting glucose and insulin-based indices are commonly used to evaluate insulin resistance-related alterations, they may not fully capture the complexity of glucose-lipid metabolic disturbances, particularly in older adults. Markers integrating multiple metabolic domains may therefore provide complementary information regarding individual metabolic profiles.

The triglyceride–glucose (TyG) index has emerged as a surrogate marker reflecting alterations in glucose and lipid metabolism and has been increasingly used in epidemiological studies investigating insulin resistance-related phenotypes [20,21]. Unlike fasting insulin-based indices, TyG integrates fasting triglyceride and glucose concentrations and may better reflect disturbances of glucose-lipid homeostasis. Importantly, TyG should be considered a surrogate marker of metabolic alterations rather than a diagnostic definition of insulin resistance.

Lipid-related biomarkers such as circulating non-esterified fatty acids (NEFA) and the atherogenic index of plasma (AIP) may provide complementary information on metabolic dysfunction and cardiometabolic risk, reflecting alterations in fatty acid metabolism and atherogenic lipid profiles, respectively [22,23,24,25]. NEFA may indicate disturbances in fatty acid availability and adipose tissue metabolism, whereas AIP provides an integrated measure of triglyceride–HDL-related atherogenic lipid balance. Therefore, these markers may capture aspects of metabolic dysfunction beyond those reflected by fasting glucose or insulin concentrations alone.

Considering the potential role of VDR signaling in adipose tissue function and lipid metabolism, as well as the ability of TyG to capture inter-individual differences in glucose-lipid metabolism, it is relevant to examine whether associations between TyG and metabolic markers differ according to *VDR* genotype.

Therefore, the present study aimed to investigate whether the association between the TyG index, reflecting inter-individual differences in glucose–lipid metabolism, and lipid-related metabolic markers, including circulating NEFA and AIP, differs according to *VDR* rs7975232 genotype in older adults.

## 2. Results

Clinical, anthropometric, and metabolic characteristics of the study population are summarized in Table 1. The study included 95 older adults with a mean age of 62.71 ± 14.13 years, and men accounted for 58.9% of the cohort. Anthropometric parameters indicated variability in body composition, with mean BMI and WHR values of 27.45 ± 5.44 kg/m^2^ and 0.99 ± 0.10, respectively. Metabolic parameters showed inter-individual variability in glucose and lipid homeostasis, including markers of insulin resistance, lipid profile, and atherogenic risk.

The distribution of *VDR* rs7975232 genotypes and allele frequencies is shown in Table 2. The AC genotype was the most frequent (44.2%), followed by CC (33.7%) and AA (22.1%). The frequencies of the C and A alleles were 55.8% and 44.2%, respectively. Genotype distribution did not differ from Hardy–Weinberg equilibrium expectations (χ^2^ = 1.02, *p* = 0.312).

Clinical and metabolic characteristics according to *VDR* rs7975232 genotype are presented in Table 3. No statistically significant differences were observed among the AA, AC, and CC genotype groups with respect to age, sex distribution, BMI, or WHR. Similarly, no significant between-group differences were found for glucose- and insulin-related markers or lipid-related and atherogenic markers (all *p* > 0.05).

**Figure 1:**
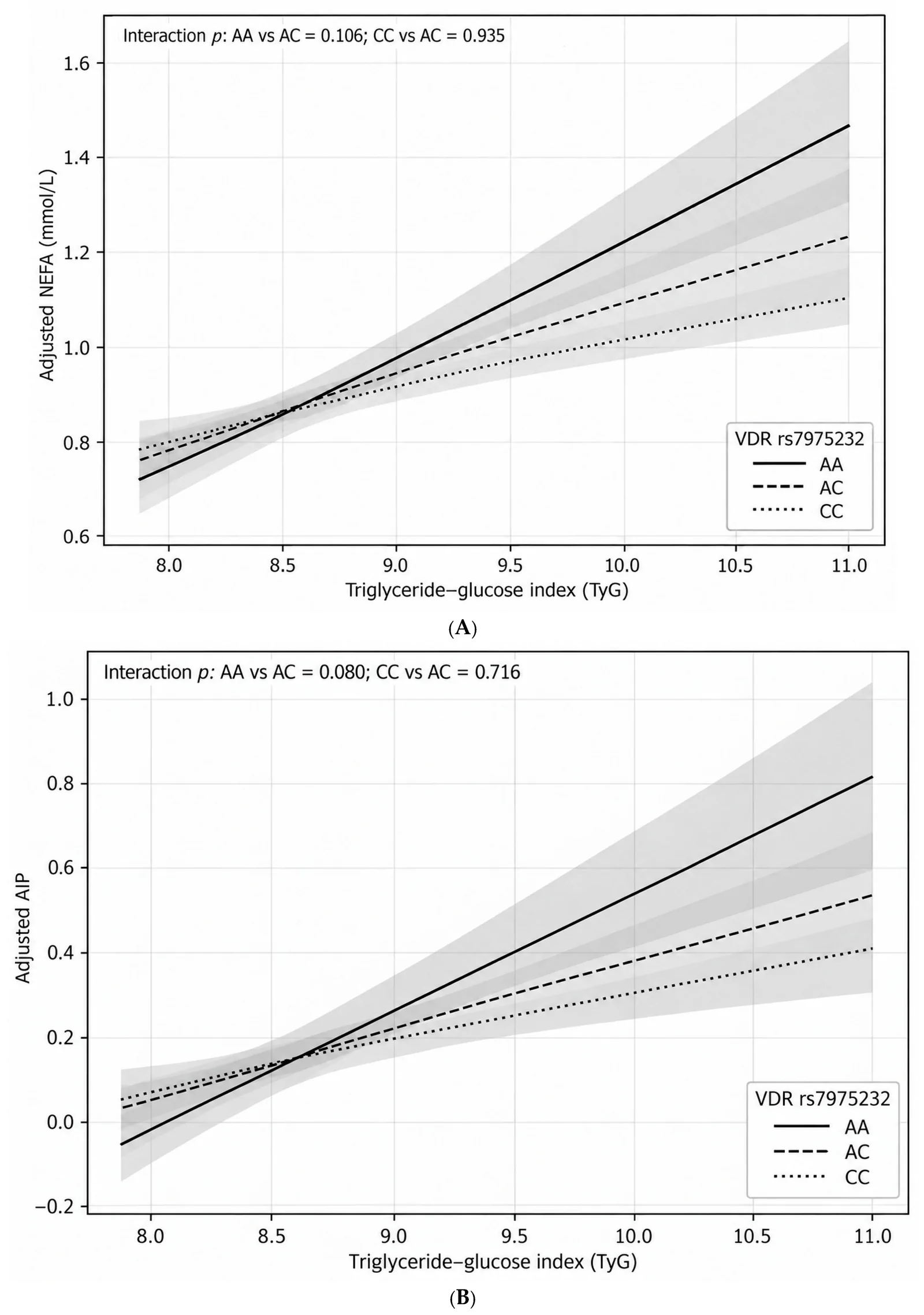
Adjusted associations between the continuous triglyceride–glucose index and NEFA and AIP according to *VDR* rs7975232 genotype. Footnotes: Adjusted predicted values of (**A**) non-esterified fatty acids (NEFA) and (**B**) atherogenic index of plasma (AIP) across the continuous TyG index according to *VDR* rs7975232 genotype. Predictions were derived from multivariable linear regression models adjusted for age, sex, and waist-to-hip ratio. Grey shaded areas represent 95% confidence intervals. The genotype × continuous TyG interactions were not statistically significant for NEFA (AA vs. AC, *p* = 0.106; CC vs. AC, *p* = 0.935) or AIP (AA vs. AC, *p* = 0.080; CC vs. AC, *p* = 0.716). Abbreviations: AIP, atherogenic index of plasma; NEFA, non-esterified fatty acids; VDR, vitamin D receptor.

Multivariable linear regression models using continuous TyG were used as the primary interaction analyses for NEFA and AIP after adjustment for age, sex, and waist-to-hip ratio. The AA and CC genotypes were entered as indicator variables, with the AC genotype used as the reference category. For NEFA, neither the AA × TyG interaction (*β* = 0.116, 95% CI: −0.025 to 0.256; *p* = 0.106) nor the CC × TyG interaction (*β* = 0.006, 95% CI: −0.137 to 0.148; *p* = 0.935) was statistically significant. Similarly, for AIP, the AA × TyG interaction did not reach statistical significance (*β* = 0.146, 95% CI: −0.018 to 0.310; *p* = 0.080), and the CC × TyG interaction was not statistically significant (*β* = −0.031, 95% CI: −0.197 to 0.136; *p* = 0.716). Adjusted genotype-specific associations across the continuous TyG range are shown in Figure 1. The full regression results are presented in Table 4.

Sensitivity analyses were performed after excluding the most influential observations identified by Cook’s distance. The AA × continuous TyG interaction remained non-significant for both NEFA (*p* = 0.191) and AIP (*p* = 0.346), and exclusion of influential observations did not reveal statistically significant genotype × TyG interactions (Supplementary Table S1).

In an additional sensitivity analysis, the interaction models were further adjusted for antidiabetic and lipid-lowering medication use. The AA × TyG interaction had a nominal *p*-value < 0.05 for AIP (*β* = 0.168, 95% CI 0.009–0.327; *p* = 0.038), but not for NEFA (*p* = 0.094), log-transformed fasting insulin (*p* = 0.216), fasting glucose (*p* = 0.234), or log(HOMA-IR) (*p* = 0.421). None of the medication-adjusted genotype × TyG interaction terms remained statistically significant after BH-FDR correction (Supplementary Table S2).

As a sensitivity analysis, TyG was dichotomized at 8.5 to examine whether the interaction pattern was consistent with the continuous-TyG models. In these categorical analyses, the AA genotype × higher TyG category interaction had nominal *p*-values < 0.05 for NEFA (*β* = 0.214, 95% CI: 0.043 to 0.386; *p* = 0.015) and AIP (*β* = 0.214, 95% CI: 0.020 to 0.407; *p* = 0.031), whereas the corresponding CC genotype interactions were not statistically significant (Supplementary Table S3). Because subgroup sizes were limited, these findings were considered exploratory. The numbers of participants in the genotype × TyG category subgroups were: AA, 8 with TyG < 8.5 and 13 with TyG ≥ 8.5; AC, 19 and 23, respectively; and CC, 10 and 22, respectively.

**Figure 2:**
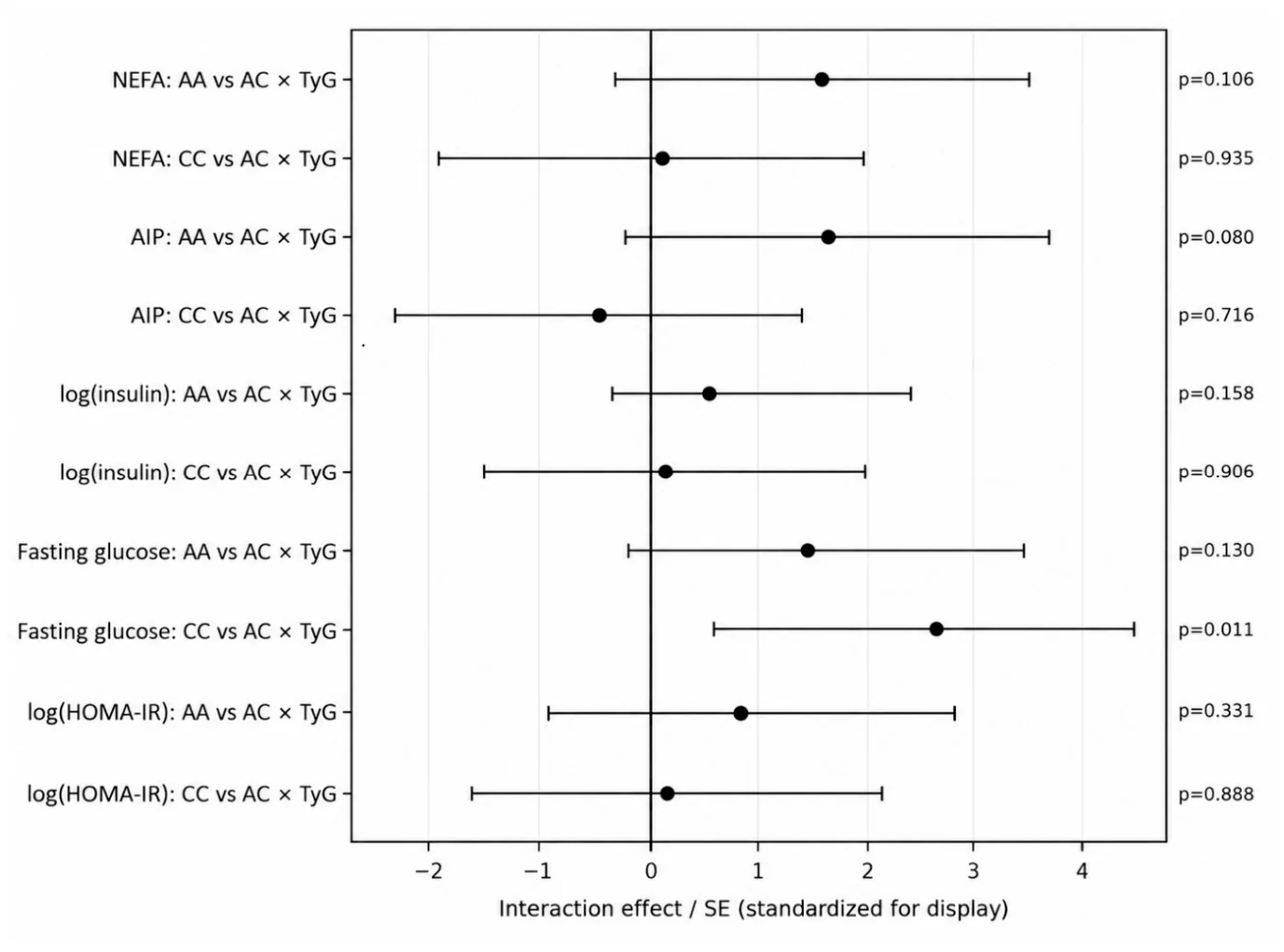
*VDR* rs7975232 genotype × continuous TyG interaction estimates across metabolic outcomes. Footnotes: Forest plot showing regression coefficients and 95% confidence intervals for genotype × continuous TyG interaction terms from multivariable linear regression models adjusted for age, sex, and waist-to-hip ratio. The AC genotype served as the reference category. Estimates are shown for NEFA, AIP, log-transformed fasting insulin, fasting glucose, and log-transformed HOMA-IR. Nominal *p*-values are displayed for each interaction contrast. None of the genotype × TyG interactions remained statistically significant after Benjamini–Hochberg false-discovery-rate correction.

Additional multivariable regression analyses using log-transformed fasting insulin, fasting glucose, and log-transformed HOMA-IR as dependent variables were performed with continuous TyG and are presented in Supplementary Table S4. A nominal genotype × TyG interaction was observed for fasting glucose (CC × TyG), whereas the genotype × TyG interaction terms for log-transformed fasting insulin and log-transformed HOMA-IR were not statistically significant. Adjusted genotype-specific associations across the continuous TyG range are shown for log-transformed fasting insulin in Supplementary Figure S1 and for fasting glucose in Supplementary Figure S2. Interaction effect estimates across all five metabolic outcomes (NEFA, AIP, log-transformed fasting insulin, fasting glucose, and log-transformed HOMA-IR) are summarized in Figure 2. However, none of these interaction terms remained statistically significant after Benjamini–Hochberg false-discovery-rate correction across the six interaction tests reported in Supplementary Table S4. These analyses were considered exploratory.

Characteristics of participants according to TyG category (TyG ≥ 8.5 vs. TyG < 8.5) are presented in Supplementary Table S5. Participants with TyG ≥ 8.5 showed higher glucose concentrations, HOMA-IR values, triglyceride levels, NEFA concentrations, and AIP values compared with those with TyG < 8.5. This categorization was used for descriptive and sensitivity analyses only and was not the primary specification of TyG in the interaction models.

## 3. Discussion

The present study investigated whether the *VDR* rs7975232 genotype was associated with heterogeneity in the relationship between TyG and selected lipid-related metabolic outcomes, including circulating NEFA concentrations and AIP, a marker reflecting the balance between triglyceride-rich lipoproteins and HDL cholesterol. In the primary analyses using continuous TyG, genotype × TyG interactions for NEFA and AIP did not reach statistical significance. In the sensitivity analysis using TyG dichotomized at 8.5, nominal AA genotype × TyG category interactions were observed for both NEFA concentrations and AIP. Importantly, rs7975232 genotype alone was not associated with differences in baseline metabolic characteristics, including TyG, NEFA, or AIP values. These findings indicate no clear independent association of rs7975232 with metabolic phenotype or genotype-dependent heterogeneity in the primary continuous-TyG analyses; the nominal findings from the categorical sensitivity analysis should be considered exploratory. Such context-dependent patterns may nevertheless be relevant to the complex genetic architecture of metabolic traits, in which associations involving genetic variants may differ according to metabolic characteristics rather than being reflected in uniform baseline differences [26].

Vitamin D–VDR signaling provides a biological context for considering potential associations between *VDR* variation and metabolic traits. The vitamin D receptor is expressed in multiple metabolically active tissues, including adipose tissue, skeletal muscle, and pancreatic β-cells, and has been implicated in pathways involved in insulin sensitivity, inflammation, adipocyte function, and lipid metabolism [27,28]. Although the functional consequences of the *VDR* rs7975232 polymorphism remain incompletely characterized, this variant has been investigated in relation to metabolic traits, vitamin D-related pathways, and chronic metabolic disorders [29,30]. However, the functional significance of rs7975232 remains uncertain, and associations involving this variant may reflect linkage disequilibrium with other functional *VDR* variants rather than a direct biological effect of rs7975232. Vitamin D status was assessed in this cohort in our previous study [31] but was not evaluated as an effect modifier in the present analysis. Therefore, the observed associations should not be interpreted as depending on vitamin D status or as being mediated specifically through altered vitamin D–VDR signaling. Given the cross-sectional design and the absence of statistically significant interactions after correction for multiple testing, the present findings should be considered hypothesis-generating rather than mechanistic evidence.

Interestingly, the differing patterns observed across fasting glucose, log-transformed insulin, and HOMA-IR in the continuous-TyG models may reflect the distinct metabolic information captured by these measures. HOMA-IR is a fasting, model-derived surrogate influenced predominantly by basal glucose-insulin homeostasis and does not provide a direct measure of peripheral insulin sensitivity. The TyG index incorporates both fasting triglyceride and glucose concentrations and provides complementary information on glucose-lipid metabolic status [32,33]. This distinction may be relevant in older populations, in whom metabolic abnormalities may be present despite normal fasting glucose concentrations and may be influenced by age-related physiological changes [34,35]. Nevertheless, differences between HOMA-IR- and TyG-based analyses should not be interpreted as demonstrating superior detection of genotype-dependent effects by either index.

In the present cohort, HOMA-IR showed substantial inter-individual variability, consistent with the metabolic heterogeneity of the study population. Age-related alterations, including reduced skeletal muscle mass and altered adiposity distribution, may influence fasting insulin resistance estimates and contribute to variability in metabolic assessment among older adults [36,37]. Accordingly, the differing genotype × TyG interaction patterns observed for HOMA-IR, fasting glucose, and log-transformed insulin may reflect differences in the metabolic information represented by these measures, although this interpretation remains speculative.

The supplementary analyses provide additional descriptive context. Participants in the higher TyG category had higher BMI, HOMA-IR, and NEFA values; however, these differences are descriptive and do not establish independent associations. The observed differences in fasting glucose and triglyceride concentrations are inherently related to the definition of the TyG index, as both variables are included in its calculation. Similarly, the higher AIP values should be interpreted cautiously because triglycerides are incorporated into both TyG and AIP, resulting in mathematical coupling between the two indices (Supplementary Table S5). Together with the differing interaction patterns across glucose- and insulin-related measures (Supplementary Table S4), these observations provide additional context for the exploratory genotype × TyG findings.

Such heterogeneity in genotype-related associations may be particularly relevant in older adults, as ageing may alter gene-environment interactions and contribute to inter-individual differences in metabolic disturbances [38]. The metabolic phenotype in later life results from the interplay between genetic background, physiological ageing, lifestyle-related factors, and comorbid conditions, which may contribute to substantial inter-individual metabolic variability [39].

Further longitudinal studies in larger cohorts are required to determine whether *VDR*-related genetic variation is associated with longitudinal differences in metabolic trajectories and to clarify the biological basis of the patterns observed in the present exploratory analysis.

Several limitations should be acknowledged. First, the cross-sectional design of the study does not allow conclusions regarding causality or temporal relationships between *VDR* rs7975232 genotype, TyG-related metabolic characteristics, and lipid-related outcomes. Second, the relatively small sample size and the number of participants within individual genotype groups may have limited the statistical power to detect weaker interaction effects, particularly for the AA genotype. Therefore, the observed interaction patterns should be considered exploratory and require confirmation in larger cohorts. Third, TyG was dichotomized at 8.5 only in sensitivity analyses for analytical stratification and should not be interpreted as a diagnostic criterion for insulin resistance. Although TyG may provide additional information regarding glucose–lipid metabolic alterations, it remains a surrogate marker and does not replace direct assessment of insulin sensitivity. Additionally, because both TyG and AIP incorporate triglyceride concentrations, mathematical coupling may partly contribute to the observed association between these indices; therefore, findings involving AIP should be interpreted cautiously. Medication use, comorbid conditions, and lifestyle-related factors remain potential sources of residual confounding. Several relevant characteristics of this cohort, including dietary intake, smoking status, polypharmacy, neuroleptic medication use, vitamin D supplementation, and C-reactive protein concentrations, were assessed and have been reported previously [31], but were not included as covariates in the present statistical models. Antidiabetic and lipid-lowering medication use was included in sensitivity-adjusted models because of its direct relevance to the glucose- and lipid-related outcomes under investigation. This adjustment did not materially alter the overall interpretation. However, detailed information on additional comorbidities, disease duration, and medication dose and duration was not comprehensively available, and residual clinical and lifestyle-related confounding therefore cannot be excluded. Although vitamin D status was assessed previously in this cohort, evaluation of vitamin D status as an effect modifier was beyond the scope of the present study. Accordingly, the present findings should not be interpreted as evidence that the observed associations depend on vitamin D status or are mediated specifically through altered vitamin D–VDR signaling. The functional significance of rs7975232 remains uncertain, and associations involving this variant may also reflect linkage disequilibrium with other functional *VDR* variants rather than a direct effect of rs7975232. Because haplotype and linkage disequilibrium analyses were not available, this possibility could not be examined in the present study. The relative homogeneity of the study population may reduce some sources of population heterogeneity; nevertheless, potential population stratification cannot be fully excluded in the absence of genome-wide ancestry information. External replication in larger independent cohorts is therefore warranted. Finally, the study population consisted of older adults with a specific clinical background, which may limit the generalizability of the findings to younger populations or individuals with different health characteristics.

## 4. Materials and Methods

### 4.1. Study Population

A total of 95 residents of a long-term care facility were enrolled in the study (56 men and 39 women). Eligible participants were aged ≥55 years and had been living in the facility for at least three months before study enrolment. Exclusion criteria were: acute infectious conditions, active cancer, and autoimmune disorders.

Participants lived in the same long-term care facility, received meals provided by the facility, and had shared schedules of organized daily activities. However, these common institutional conditions do not imply comparable individual dietary intake, actual levels of mobility and physical activity, sunlight exposure, health status, medication use, or other lifestyle-related factors.

All participants underwent a comprehensive clinical evaluation, including medical history assessment, physical examination performed by an internal medicine specialist, anthropometric measurements, and laboratory testing. A more detailed clinical and lifestyle characterization of this cohort, including dietary intake, smoking status, medication use, vitamin D supplementation, and inflammatory status, has been reported previously [31].

### 4.2. Measures

Anthropometric parameters were assessed according to a standardized protocol. Body weight and height were measured using an electronic personal scale (Mensor WE150P1, JAWAG, Morawica, Poland) and a wall-mounted stadiometer (Comed S.C., Koszalin, Poland), respectively. Body mass index (BMI) was calculated as body weight (kg) divided by height squared (m^2^). Waist and hip circumferences were measured using a non-stretch measuring tape, and the waist-to-hip ratio (WHR) was subsequently calculated.

Blood samples were collected in the morning between 08:00 and 08:30 after an overnight fast of at least eight hours. Serum was separated by centrifugation and stored at −70 °C until biochemical analyses. All standards and serum samples were analyzed in duplicate.

Fasting serum concentrations of glucose (cat. no. G7521), total cholesterol (TC; cat. no. C7510), high-density lipoprotein cholesterol (HDL-C; cat. no. H6421), low-density lipoprotein cholesterol (LDL-C; cat. no. L6410), triglycerides (TG; cat. no. T7531) were measured using routine enzymatic colorimetric assays (Pointe Scientific, Canton, MI, USA) according to the manufacturer’s instructions. The optical density of the samples was determined using a Synergy 2 and/or Synergy H1 microplate reader (BioTek, Winooski, VT, USA).

Serum non-esterified fatty acids (NEFA) were determined using the NEFA-HR(2) enzymatic colorimetric assay kit (FUJIFILM Wako Pure Chemical Corporation, Osaka, Japan), according to the manufacturer’s instructions.

The atherogenic index of plasma was calculated using the following formula:

$$AIP=log10\;\left(\frac{TG(mmol/L)}{HDL-C(mmol/L)}\right)$$

Serum insulin concentrations were determined using a commercially available enzyme-linked immunosorbent assay (ELISA) based on the sandwich principle (Qayee Biotechnology Co., Shanghai, China; Cat. No. QY-E00519), according to the manufacturer’s protocol. Insulin resistance was estimated using the Homeostasis Model Assessment of Insulin Resistance (HOMA-IR):

$$HOMA-IR=\frac{fastingglucose(mmol/L)\times fastinginsulin(µIU/mL)}{22.5}$$

The triglyceride–glucose (TyG) index was calculated using the following formula:

$$TyG=ln\left(\frac{TG(mg/dL)\times glucose(mg/dL)}{2}\right)$$

To convert glucose and triglyceride concentrations from SI units (mmol/L) into conventional units (mg/dL) for TyG calculation, the following conversion factors were used: 1 mmol/L glucose = 18 mg/dL and 1 mmol/L triglycerides = 88.57 mg/dL.

For the primary interaction analyses, TyG was analyzed as a continuous variable. In addition, TyG was dichotomized at 8.5 in exploratory sensitivity analyses to stratify participants into lower and higher TyG categories. The value of 8.5 was selected for analytical stratification based on TyG levels previously reported in adult and older populations [32,40], and was not considered a diagnostic criterion for insulin resistance or a threshold derived from the present cohort.

### 4.3. VDR rs7975232 Genotype Determination

Genomic DNA was extracted from peripheral blood samples collected in EDTA-containing tubes using a commercial DNA isolation kit (High Pure PCR Template Preparation Kit; Roche Diagnostics, Basel, Switzerland) in accordance with the manufacturer’s recommendations. DNA concentration and purity were assessed before genotyping procedures.

The *VDR* rs7975232 (ApaI, A/C) polymorphism was analyzed using the LightSNiP rs7975232 *VDR* Apa assay (TIB MOLBIOL, Berlin, Germany) performed on a LightCycler^®^ 480 Real-Time PCR System (Roche Diagnostics, Basel, Switzerland). Genotype determination was based on allele-specific amplification followed by melting curve analysis using the sequence-specific primers and hybridization probes provided by the manufacturer.

Each PCR reaction was conducted in a total volume of 20 μL, containing 2.0 μL LightCycler^®^ 480 FastStart DNA Master HybProbe mix (Roche Diagnostics, Mannheim, Germany), 1.6 μL MgCl_2_ (25 mM), 1.0 μL primer/probe mixture, 1.0–5.0 μL genomic DNA (approximately 50 ng), and PCR-grade water to obtain the final reaction volume.

Amplification was carried out with an initial denaturation step at 95 °C for 10 min, followed by 45 cycles of denaturation at 95 °C for 10 s, annealing at 60 °C for 10 s, and extension at 72 °C for 15 s. Allelic discrimination was performed using melting curve analysis consisting of denaturation at 95 °C for 30 s, cooling to 40 °C for 2 min, and subsequent gradual temperature increase to 75 °C with continuous fluorescence measurement. Allele identification was based on the characteristic melting profiles generated by the assay. Characteristic allele-specific melting peaks were interpreted at approximately 62.8 °C for the C allele and 69.9 °C for the A allele. These melting temperatures are consistent with previously published rs7975232 genotyping studies by our group using real-time PCR and melting-curve analysis [41,42].

Negative controls were included in each PCR run to monitor potential contamination. Genotyping reproducibility was confirmed by repeating the analysis in 10 randomly selected samples (10.5% of the study sample), with complete agreement between repeated measurements.

### 4.4. Statistics

Continuous variables were presented as mean ± standard deviation (SD) or median with interquartile range (IQR), depending on data distribution. Categorical variables were expressed as numbers and percentages. The distribution of continuous variables was assessed using the Shapiro–Wilk test. Variables that did not meet the assumptions of normal distribution were analyzed using non-parametric methods. Because fasting insulin showed a right-skewed distribution, it was log-transformed for the regression analyses. The Hardy–Weinberg equilibrium (HWE) for the *VDR* rs7975232 genotype distribution was evaluated using an online HWE calculator (https://wpcalc.com/en/equilibrium-hardy-weinberg/, accessed on 4 August 2026).

Differences in clinical, anthropometric, and metabolic characteristics between rs7975232 genotype groups (AA, AC, and CC) were assessed using one-way analysis of variance (ANOVA) for normally distributed variables or the Kruskal–Wallis test for non-normally distributed variables. Categorical variables were compared using the chi-square test.

To investigate whether the association between the TyG index and lipid-related metabolic markers differed according to *VDR* rs7975232 genotype, multivariable linear regression models were constructed separately for NEFA and AIP as dependent variables. Additional genotype × TyG interaction models were constructed for log-transformed fasting insulin, fasting glucose, and log-transformed HOMA-IR and are reported in the Supplementary Materials. In the primary analyses, TyG was mean-centered and entered as a continuous variable, and genotype was modeled codominantly, with categories AA and CC coded as indicator variables and AC serving as the reference category because it was the most frequent genotype in the study population. The choice of AC as the reference category was a parameterization decision and did not imply a biologically defined reference state. Genotype × TyG interaction terms were included, with adjustment for age, sex, and WHR. For NEFA and AIP, exploratory sensitivity analyses were additionally performed using TyG categorized as <8.5 or ≥8.5. Regression coefficients (β) with 95% confidence intervals (CI) were reported. To account for multiple testing, Benjamini–Hochberg false-discovery-rate (BH-FDR) correction was applied separately to the four primary genotype × TyG interaction tests for NEFA and AIP (two outcomes × two genotype contrasts) and to the six exploratory interaction tests reported in Supplementary Table S4 (three outcomes × two genotype contrasts). A two-sided nominal *p*-value < 0.05 was considered nominally significant, whereas FDR-adjusted *p*-values were used to assess statistical significance after correction for multiple testing.

As an additional sensitivity analysis, the genotype × continuous TyG models for NEFA, AIP, log-transformed fasting insulin, fasting glucose, and log-transformed HOMA-IR were further adjusted for antidiabetic and lipid-lowering medication use. These medication variables were selected a priori as the pharmacological factors most directly related to glucose and lipid metabolism. Benjamini–Hochberg false-discovery-rate correction was applied across the 10 genotype × TyG interaction tests evaluated in these medication-adjusted models.

Regression diagnostics included assessment of standardized residuals, Cook’s distance, Mahalanobis distance, and multicollinearity. Influential observations identified by Cook’s distance were examined in sensitivity analyses by repeating the primary models after their exclusion.

All statistical analyses were performed using STATISTICA 13 software (TIBCO Software Inc., Palo Alto, CA, USA) for Windows (Microsoft Corporation, Redmond, WA, USA).

## 5. Conclusions

In this exploratory cross-sectional study, *VDR* rs7975232 was not independently associated with the assessed metabolic traits. The primary analyses did not provide robust evidence of genotype-dependent heterogeneity in associations with TyG. Nevertheless, the findings from the exploratory sensitivity analysis suggest that potential differences in TyG–metabolic associations according to *VDR* genotype warrant further investigation. These observations are hypothesis-generating and require confirmation in larger independent cohorts.

## Figures and Tables

**Table 1:** Clinical and metabolic characteristics of the study population (*n* = 95).

Variable	Mean ± SD	Median (IQR)
**Demographic and anthropometric characteristics**		-
Age (years)	62.71 ± 14.13	-
Male, *n* (%)	56 (58.95%)	-
Female, *n* (%)	39 (41.05%)	-
WHR	0.99 ± 0.10	-
BMI (kg/m^2^)	27.45 ± 5.44	-
**Glucose and lipid metabolism**		
Glucose (mmol/L)	-	4.23 (3.80–5.33)
Insulin (µIU/mL)	-	18.16 (16.85–25.05)
HOMA-IR	-	3.61 (3.09–7.31)
TyG	-	8.63 (8.37–8.99)
TC (mmol/L)	-	5.23 (4.66–6.18)
TG (mmol/L)	-	1.54 (1.27–1.86)
LDL-C (mmol/L)	-	2.68 (2.31–3.14)
HDL-C (mmol/L)	-	1.01 (0.88–1.19)
NEFA (mmol/L)	-	0.88 (0.82–1.00)
AIP	-	0.18 (0.06–0.31)

Footnotes: Bold indicates section headers. Data are presented as mean ± SD for normally distributed variables, median (IQR) for non-normally distributed variables, or n (%) for categorical variables. Abbreviations: AIP, atherogenic index of plasma; BMI, body mass index; HDL-C, high-density lipoprotein cholesterol; HOMA-IR, homeostasis model assessment of insulin resistance; IQR, interquartile range; LDL-C, low-density lipoprotein cholesterol; NEFA, non-esterified fatty acids; SD, standard deviation; TC, total cholesterol; TG, triglycerides; TyG, triglyceride–glucose index; WHR, waist-to-hip ratio.

**Table 2:** Distribution of *VDR* rs7975232 genotypes and Hardy–Weinberg equilibrium.

Genotype/Allele	Observed *n* (%)	Expected *n* (%)
AA	21 (22.1%)	18.6 (19.6%)
AC	42 (44.2%)	46.9 (49.4%)
CC	32 (33.7%)	29.6 (31.2%)
A allele	84 (44.2%)	-
C allele	106 (55.8%)	-

Hardy–Weinberg equilibrium: χ^2^ = 1.02; *p* = 0.312.

**Table 3:** Demographic, anthropometric, glucose–lipid, and cardiometabolic characteristics according to *VDR* rs7975232 genotype.

	AA *n* = 21	AC *n* = 42	CC *n* = 32	Test	*p*
**Demographic and anthropometric characteristics**					
Age (years)	58.62 ± 11.36	62.33 ± 15.52	65.88 ± 13.49	F = 1.72	0.184
Male, *n* (%)	13 (61.9)	23 (54.8)	20 (62.5)	χ^2^ = 0.55	0.761
Female, *n* (%)	8 (38.1)	19 (45.2)	12 (37.5)		
BMI (kg/m^2^)	27.88 ± 4.66	26.72 ± 5.74	28.13 ± 5.54	F = 0.68	0.506
WHR	1.02 ± 0.12	0.97 ± 0.10	0.98 ± 0.09	F = 1.37	0.259
**Glucose and insulin-related markers**					
Glucose (mmol/L)	4.30 (4.10–5.50)	4.03 (3.67–4.75)	4.29 (3.95–6.34)	H = 4.77	0.092
Insulin (µIU/mL)	18.83 (17.07–41.57)	18.24 (16.69–27.66)	18.06 (16.64–21.53)	H = 1.18	0.554
HOMA-IR	4.42 (3.09–10.04)	3.35 (2.89–6.07)	3.83 (3.22–7.01)	H = 1.99	0.369
TyG index	8.63 (8.39–9.10)	8.52 (8.26–8.80)	8.64 (8.39–9.21)	H = 2.28	0.320
**Lipid-related and atherogenic markers**					
TC (mmol/L)	5.15 (4.89–6.56)	5.24 (4.78–6.32)	5.19 (4.62–6.01)	H = 0.47	0.789
TG (mmol/L)	1.63 (1.27–1.87)	1.51 (1.28–1.80)	1.59 (1.32–1.92)	H = 0.50	0.777
LDL-C (mmol/L)	2.46 (2.26–3.55)	2.72 (2.34–3.13)	2.65 (2.31–3.00)	H = 0.74	0.690
HDL-C (mmol/L)	1.00 (0.89–1.14)	1.01 (0.93–1.29)	1.02 (0.87–1.15)	H = 0.76	0.683
NEFA (mmol/L)	0.92 (0.83–1.09)	0.87 (0.82–0.96)	0.88 (0.79–0.99)	H = 1.95	0.376
AIP	0.15 (0.06–0.35)	0.17 (0.05–0.27)	0.18 (0.10–0.30)	H = 0.61	0.736

Footnotes: Bold indicates section headers. Data are presented as mean ± SD for normally distributed variables, median (IQR) for non-normally distributed variables, or n (%) for categorical variables. Differences between genotype groups were assessed using one-way ANOVA (F), the Kruskal–Wallis test (H), or the chi-square test (χ^2^), as appropriate. The *p*-value for sex refers to the overall sex distribution across genotype groups. Abbreviations: AIP, atherogenic index of plasma; BMI, body mass index; HDL-C, high-density lipoprotein cholesterol; HOMA-IR, homeostasis model assessment of insulin resistance; IQR, interquartile range; LDL-C, low-density lipoprotein cholesterol; NEFA, non-esterified fatty acids; SD, standard deviation; TC, total cholesterol; TG, triglycerides; TyG, triglyceride–glucose index; VDR, vitamin D receptor; WHR, waist-to-hip ratio.

**Table 4:** Multivariable linear regression models assessing the interaction between *VDR* rs7975232 genotype and continuous TyG in relation to NEFA and atherogenic index of plasma (AIP).

Predictor	NEFA *β* (95% CI)	Nominal *p*	BH-FDR *p*	AIP *β* (95% CI)	Nominal *p*	BH-FDR *p*
Genotype AA vs. AC	0.026 (−0.049 to 0.101)	0.491	—	0.006 (−0.082 to 0.093)	0.894	—
Genotype CC vs. AC	−0.013 (−0.079 to 0.054)	0.704	—	−0.003 (−0.081 to 0.075)	0.935	—
Centered TyG	0.160 (0.043 to 0.276)	0.008	—	0.177 (0.041 to 0.313)	0.011	—
AA × centered TyG	0.116 (−0.025 to 0.256)	0.106	0.212	0.146 (−0.018 to 0.310)	0.080	0.212
CC × centered TyG	0.006 (−0.137 to 0.148)	0.935	0.935	−0.031 (−0.197 to 0.136)	0.716	0.935
Age	−0.001 (−0.003 to 0.002)	0.642	—	−0.000 (−0.003 to 0.003)	0.931	—
Sex	0.060 (−0.004 to 0.125)	0.065	—	0.076 (0.001 to 0.151)	0.047	—
WHR	−0.285 (−0.599 to 0.030)	0.076	—	0.208 (−0.160 to 0.575)	0.264	—

Footnotes: Data are presented as unstandardized regression coefficients (β) with 95% confidence intervals (CI). TyG was mean-centered before construction of genotype × TyG interaction terms. Models were adjusted for age, sex, and waist-to-hip ratio (WHR). The AC genotype served as the reference category. BH-FDR *p*-values are shown only for genotype × TyG interaction terms and were calculated using the Benjamini–Hochberg procedure across the four interaction tests reported in Table 4 (two outcomes × two genotype contrasts). None of the interaction terms remained statistically significant after FDR correction. Abbreviations: AIP, atherogenic index of plasma; BH-FDR, Benjamini–Hochberg false-discovery rate; CI, confidence interval; NEFA, non-esterified fatty acids; TyG, triglyceride–glucose index; VDR, vitamin D receptor; WHR, waist-to-hip ratio.

## Data Availability

The data presented in this study are available from the corresponding author upon reasonable request. The data are not publicly available due to privacy concerns.

## Supplementary Materials

The following supporting information can be downloaded at https://www.mdpi.com/article/10.3390/ijms27209252/s1.

## References

[1] Bou Matar D., Zhra M., Nassar W.K., Altemyatt H., Naureen A., Abotouk N., Elahi M.A., Aljada A. (2025). Adipose tissue dysfunction disrupts metabolic homeostasis: Mechanisms linking fat dysregulation to disease. Front. Endocrinol..

[2] Liu J., Huang Q., Liu F. (2025). Fat talks first: How adipose tissue sets the pace of aging?. Life Med..

[3] Liu Z., Wu K.K.L., Jiang X., Xu A., Cheng K.K.Y. (2020). The role of adipose tissue senescence in obesity- and ageing-related metabolic disorders. Clin. Sci..

[4] Hsu S.W., Yen C.F., Hung W.J., Lin L.P., Wu C.L., Lin J.D. (2012). The risk of metabolic syndrome among institutionalized adults with intellectual disabilities. Res. Dev. Disabil..

[5] Zhang K., Ma Y., Luo Y., Song Y., Xiong G., Ma Y., Sun X., Kan C. (2023). Metabolic diseases and healthy aging: Identifying environmental and behavioral risk factors and promoting public health. Front. Public Health.

[6] Yousri N.A., Kastenmüller G., Gieger C., Shin S.Y., Erte I., Menni C., Peters A., Meisinger C., Mohney R.P., Illig T. (2014). Long term conservation of human metabolic phenotypes and link to heritability. Metabolomics.

[7] Fuentes-Barría H., Aguilera-Eguía R., Alarcón-Rivera M., Angarita-Davila L., Flores-Fernández C. (2026). Vitamin D and metabolic dysfunction-associated steatotic liver disease: Molecular mechanisms and clinical implications—A narrative review. Int. J. Mol. Sci..

[8] Szymczak-Pajor I., Drzewoski J., Śliwińska A. (2020). The molecular mechanisms by which vitamin D prevents insulin resistance and associated disorders. Int. J. Mol. Sci..

[9] Norman A.W. (2008). From vitamin D to hormone D: Fundamentals of the vitamin D endocrine system essential for good health. Am. J. Clin. Nutr..

[10] Park C.Y., Han S.N. (2021). The role of vitamin D in adipose tissue biology: Adipocyte differentiation, energy metabolism, and inflammation. J. Lipid Atheroscler..

[11] Bennour I., Haroun N., Sicard F., Mounien L., Landrier J.F. (2022). Recent insights into vitamin D, adipocyte, and adipose tissue biology. Obes. Rev..

[12] Abouzid M., Główka F., Kagan L., Karaźniewicz-Łada M. (2022). Vitamin D metabolism gene polymorphisms and their associated disorders: A literature review. Curr. Drug Metab..

[13] Al-Daghri N.M., Mohammed A.K., Al-Attas O.S., Ansari M.G.A., Wani K., Hussain S.D., Sabico S., Tripathi G., Alokail M.S. (2017). Vitamin D receptor gene polymorphisms modify cardiometabolic response to vitamin D supplementation in T2DM patients. Sci. Rep..

[14] Rashidi F., Ostadsharif M. (2021). Association of *VDR* gene ApaI polymorphism with obesity in Iranian population. Biomedica.

[15] Alathari B.E., Sabta A.A., Kalpana C.A., Vimaleswaran K.S. (2020). Vitamin D pathway-related gene polymorphisms and their association with metabolic diseases: A literature review. J. Diabetes Metab. Disord..

[16] Han F.-F., Lv Y.-L., Gong L.-L., Liu H., Wan Z.-R., Liu L.-H. (2017). *VDR* gene variation and insulin resistance related diseases. Lipids Health Dis..

[17] Totonchi H., Rezaei R., Noori S., Azarpira N., Mokarram P., Imani D. (2021). Vitamin D receptor gene polymorphisms and the risk of metabolic syndrome (MetS): A meta-analysis. Endocr. Metab. Immune Disord. Drug Targets.

[18] Karonova T., Grineva E., Belyaeva O., Bystrova A., Jude E.B., Andreeva A., Kostareva A., Pludowski P. (2018). Relationship between vitamin D status and vitamin D receptor gene polymorphisms with markers of metabolic syndrome among adults. Front. Endocrinol..

[19] Jin T., Lu W., Gong X., Zhou J., Wu F. (2021). Association of vitamin D receptor polymorphisms with metabolic syndrome-related components: A cross-sectional study. J. Clin. Lab. Anal..

[20] Chen M., Guo J., Shangguan Y., Sun Z., He X., Tu Q., Yan Q. (2025). Association of the triglyceride-glucose index combined with a body shape index with all-cause and cardiovascular mortality in individuals with cardiovascular-kidney-metabolic syndrome stage 0–3: Findings from two prospective cohorts. Cardiovasc. Diabetol..

[21] Zhao Q., Zhang T.Y., Cheng Y.J., Ma Y., Xu Y.K., Yang J.Q., Zhou Y.J. (2021). Triglyceride-glucose index as a surrogate marker of insulin resistance for predicting cardiovascular outcomes in nondiabetic patients with non-ST-segment elevation acute coronary syndrome undergoing percutaneous coronary intervention. J. Atheroscler. Thromb..

[22] Stefan N., Stumvoll M., Bogardus C., Tataranni P.A. (2003). Elevated plasma nonesterified fatty acids are associated with deterioration of acute insulin response in IGT but not NGT. Am. J. Physiol. Endocrinol. Metab..

[23] Djoussé L., Biggs M.L., Ix J.H., Kizer J.R., Lemaitre R.N., Sotoodehnia N., Zieman S.J., Mozaffarian D., Tracy R.P., Mukamal K.J. (2012). Nonesterified fatty acids and risk of sudden cardiac death in older adults. Circ. Arrhythm. Electrophysiol..

[24] Dobiásová M., Frohlich J. (2001). The plasma parameter log (TG/HDL-C) as an atherogenic index: Correlation with lipoprotein particle size and esterification rate in apoB-lipoprotein-depleted plasma (FER(HDL)). Clin. Biochem..

[25] Rashidian P., Mirchandani M., Somu K.P., Jala S.R.A., Mahapatro A., Hashemi S.M., Nasrollahizadeh A., Joukar F., Eshraghi R., Letafatkar N. (2025). Association between atherogenic index of plasma and various metabolic conditions: An umbrella review on meta-analyses. BMC Cardiovasc. Disord..

[26] Barroso I., McCarthy M.I. (2019). The genetic basis of metabolic disease. Cell.

[27] Argano C., Mirarchi L., Amodeo S., Orlando V., Torres A., Corrao S. (2023). The role of vitamin D and its molecular bases in insulin resistance, diabetes, metabolic syndrome, and cardiovascular disease: State of the art. Int. J. Mol. Sci..

[28] Contreras-Bolívar V., García-Fontana B., García-Fontana C., Muñoz-Torres M. (2021). Mechanisms involved in the relationship between vitamin D and insulin resistance: Impact on clinical practice. Nutrients.

[29] Fronczek M., Osadnik T., Banach M. (2023). Impact of vitamin D receptor polymorphisms in selected metabolic disorders. Curr. Opin. Clin. Nutr. Metab. Care.

[30] Santos B.R., Lecke S.B., Spritzer P.M. (2018). Apa-I polymorphism in *VDR* gene is related to metabolic syndrome in polycystic ovary syndrome: A cross-sectional study. Reprod. Biol. Endocrinol..

[31] Czyzniewski B., Chmielowiec J., Pruszynska-Oszmalek E., Hajduk-Warchoł M., Chmielowiec K., Kolodziejski P., Huzarski T., Checinska-Maciejewska Z., Kowalski M.T., Szczap E. (2025). Associations between vitamin D2 and metabolic indices in institutionalized older adults: A cross-sectional study. J. Physiol. Pharmacol..

[32] Liang D., Liu C., Wang Y. (2024). The association between triglyceride-glucose index and the likelihood of cardiovascular disease in the U.S. population of older adults aged ≥60 years: A population-based study. Cardiovasc. Diabetol..

[33] Gounden V., Devaraj S., Jialal I. (2024). The role of the triglyceride-glucose index as a biomarker of cardio-metabolic syndromes. Lipids Health Dis..

[34] Carlson O.D., David J.D., Schrieder J.M., Muller D.C., Jang H.J., Kim B.J., Egan J.M. (2007). Contribution of nonesterified fatty acids to insulin resistance in the elderly with normal fasting but diabetic 2-hour postchallenge plasma glucose levels: The Baltimore Longitudinal Study of Aging. Metabolism.

[35] Boccardi V., Sinclair A.J. (2026). Rethinking insulin resistance in aging: A reserve-oriented clinical framework. Ageing Res. Rev..

[36] Kalyani R.R., Corriere M., Ferrucci L. (2014). Age-related and disease-related muscle loss: The effect of diabetes, obesity, and other diseases. Lancet Diabetes Endocrinol..

[37] Stenholm S., Harris T.B., Rantanen T., Visser M., Kritchevsky S.B., Ferrucci L. (2008). Sarcopenic obesity: Definition, cause and consequences. Curr. Opin. Clin. Nutr. Metab. Care.

[38] Barrere-Cain R., Allard P. (2020). An understudied dimension: Why age needs to be considered when studying epigenetic-environment interactions. Epigenet. Insights.

[39] Palmer A.K., Jensen M.D. (2022). Metabolic changes in aging humans: Current evidence and therapeutic strategies. J. Clin. Investig..

[40] Kurniawan L.B. (2024). Triglyceride-glucose index as a biomarker of insulin resistance, diabetes mellitus, metabolic syndrome, and cardiovascular disease: A review. EJIFCC.

[41] Suchanecka A., Chmielowiec K., Chmielowiec J., Trybek G., Masiak J., Michałowska-Sawczyn M., Nowicka R., Grocholewicz K., Grzywacz A. (2020). Vitamin D receptor gene polymorphisms and cigarette smoking impact on oral health: A case-control study. Int. J. Environ. Res. Public Health.

[42] Larysz D., Recław R., Suchanecka A., Dziurawiec W., Tkacz R., Strońska-Pluta A., Chmielowiec K., Grzywacz A., Chmielowiec J. (2025). Vitamin D receptor rs7975232 (ApaI) variant, inflammatory markers, and patient-reported outcomes in orthopedic surgery. J. Clin. Med..

