# Supplementary material for: Interaction Analysis of VDR rs7975232 Polymorphism and the Triglyceride-Glucose Index in Relation to Metabolic Outcomes in Older Adults
Source: Int J Mol Sci. 2026 Oct 18;27(20):9252. doi: 10.3390/ijms27209252 (PMC13705050; doi:10.3390/ijms27209252)
Supplement: Supplementary file 1 [file ijms-27-09252-s001.zip › ijms-4557256-supplementary.pdf]

Table S1. Sensitivity analysis of genotype × continuous TyG interactions after exclusion of influential observations identified by Cook's distance.

Outcome	Interaction	Full Sample *β* (95% CI)	*p*	Sensitivity *β* (95% CI)	*p*	Excluded Observations
NEFA	AA vs AC × centered TyG	0.116 (−0.025 to 0.256)	0.106	0.090 (−0.046 to 0.227)	0.191	2
NEFA	CC vs AC × centered TyG	0.006 (−0.137 to 0.148)	0.935	−0.032 (−0.155 to 0.092)	0.611	2
AIP	AA vs AC × centered TyG	0.146 (−0.018 to 0.310)	0.080	0.091 (−0.100 to 0.283)	0.346	3
AIP	CC vs AC × centered TyG	−0.031 (−0.197 to 0.136)	0.716	−0.003 (−0.156 to 0.150)	0.969	3

Footnotes: Sensitivity models used the same covariate specification as the primary models (age, sex, and WHR). Influential observations were identified using Cook's distance and excluded only for this sensitivity analysis.

Abbreviations: AIP, atherogenic index of plasma; CI, confidence interval; NEFA, non-esterified fatty acids; TyG, triglyceride–glucose index; WHR, waist-to-hip ratio.

Table S2. Sensitivity analysis of genotype × continuous TyG interactions after additional adjustment for antidiabetic and lipid-lowering medication use.

Outcome	Interaction	*β*	95% CI	Nominal *p*	BH-FDR adjusted *p*
AIP	AA vs AC × centered TyG	0.168	0.009 to 0.327	0.038	0.380
AIP	CC vs AC × centered TyG	0.042	−0.126 to 0.210	0.620	0.689
NEFA	AA vs AC × centered TyG	0.122	−0.021 to 0.265	0.094	0.468
NEFA	CC vs AC × centered TyG	0.016	−0.134 to 0.167	0.830	0.830
log(fasting insulin)	AA vs AC × centered TyG	0.547	−0.326 to 1.420	0.216	0.468
log(fasting insulin)	CC vs AC × centered TyG	−0.290	−1.211 to 0.631	0.533	0.666
Fasting glucose	AA vs AC × centered TyG	10.01	−6.59 to 26.61	0.234	0.468
Fasting glucose	CC vs AC × centered TyG	11.93	−5.59 to 29.45	0.179	0.468
log(HOMA-IR)	AA vs AC × centered TyG	0.470	−0.686 to 1.626	0.421	0.666
log(HOMA-IR)	CC vs AC × centered TyG	−0.302	−1.258 to 0.654	0.531	0.666

Footnotes: Antidiabetic medications were used by 20 of 95 participants (21.1%), and lipid-lowering medications by 25 of 95 participants (26.3%). TyG was mean-centered before construction of the interaction terms. Estimates are from multivariable linear regression models adjusted for age, sex, waist-to-hip ratio (WHR), antidiabetic medication use, and lipid-lowering medication use, with the AC genotype serving as the reference category. Fasting insulin and HOMA-IR were log-transformed before analysis. BH-FDR-adjusted *p* values were calculated using the Benjamini–Hochberg procedure across the ten genotype × TyG interaction tests shown in this table.

Abbreviations: AIP, atherogenic index of plasma; BH-FDR, Benjamini–Hochberg false-discovery rate; CI, confidence interval; HOMA-IR, homeostasis model assessment of insulin resistance; NEFA, non-esterified fatty acids; TyG, triglyceride–glucose index; WHR, waist-to-hip ratio.

Table S3. Exploratory sensitivity analysis of *VDR* rs7975232 genotype × TyG category interactions in relation to NEFA and AIP.

Predictor	NEFA β (95% CI)	p	AIP β (95% CI)	p
Genotype AA vs AC	−0.285 (−0.570 to 0.001)	0.051	−0.299 (−0.622 to 0.024)	0.069
Genotype CC vs AC	−0.050 (−0.302 to 0.202)	0.692	−0.081 (−0.365 to 0.204)	0.574
TyG ≥8.5	0.064 (−0.036 to 0.163)	0.208	0.092 (−0.021 to 0.205)	0.108
AA × TyG ≥8.5	0.214 (0.043 to 0.386)	0.015	0.214 (0.020 to 0.407)	0.031
CC × TyG ≥8.5	0.036 (−0.116 to 0.188)	0.640	0.057 (−0.114 to 0.229)	0.509
Age	−0.001 (−0.003 to 0.002)	0.525	−0.000 (−0.003 to 0.003)	0.827
Sex	0.049 (−0.025 to 0.122)	0.193	0.068 (−0.015 to 0.152)	0.106
WHR	0.058 (−0.284 to 0.400)	0.736	0.575 (0.188 to 0.961)	0.004

Footnotes: Data are presented as unstandardized regression coefficients (β) with 95% confidence intervals (CI). Models were adjusted for age, sex, and waist-to-hip ratio (WHR). The AC genotype and TyG <8.5 category served as reference categories. This was an exploratory sensitivity analysis; the primary interaction analyses used continuous mean-centered TyG. Genotype × TyG category subgroup counts were: AA, 8 with TyG <8.5 and 13 with TyG ≥8.5; AC, 19 and 23, respectively; CC, 10 and 22, respectively. Abbreviations: AIP, atherogenic index of plasma; CI, confidence interval; NEFA, non-esterified fatty acids; TyG, triglyceride–glucose index; VDR, vitamin D receptor; WHR, waist-to-hip ratio.

Table S4. Genotype × continuous TyG interaction estimates for log-transformed fasting insulin, fasting glucose, and log-transformed HOMA-IR.

Outcome	Interaction	β	95% CI	Nominal p	BH-FDR adjusted p
log(fasting insulin)	AA vs AC × centered TyG	0.624	−0.247 to 1.495	0.158	0.316
log(fasting insulin)	CC vs AC × centered TyG	−0.053	−0.936 to 0.830	0.906	0.906
Fasting glucose	AA vs AC × centered TyG	14.10	−4.25 to 32.44	0.130	0.316
Fasting glucose	CC vs AC × centered TyG	24.35	5.75 to 42.94	0.011	0.066
log(HOMA-IR)	AA vs AC × centered TyG	0.580	−0.599 to 1.759	0.331	0.497
log(HOMA-IR)	CC vs AC × centered TyG	0.067	−0.875 to 1.009	0.888	0.906

Footnotes: TyG was mean-centered before construction of interaction terms. Estimates are from multivariable linear regression models adjusted for age, sex, and waist-to-hip ratio (WHR), with AC as the reference genotype. HOMA-IR and fasting insulin were log-transformed before analysis. BH-FDR-adjusted p values were calculated using the Benjamini–Hochberg procedure across the six genotype × TyG interaction tests reported in this table (three outcomes × two genotype contrasts). NEFA and AIP interaction estimates are reported separately in Table 4 and were not included in this six-test correction.

Abbreviations: AIP, atherogenic index of plasma; BH-FDR, Benjamini–Hochberg false-discovery rate; CI, confidence interval; HOMA-IR, homeostasis model assessment of insulin resistance; NEFA, non-esterified fatty acids; TyG, triglyceride–glucose index; WHR, waist-to-hip ratio

Table S5. Clinical and biochemical characteristics of study participants according to TyG category.

Variable	TyG ≥8.5 *n* = 58	TyG <8.5 *n* = 37	*p*
Demographic and anthropometric characteristics			
Age (years)	62.47 ± 13.30	63.08 ± 15.53	0.837
Male, n (%)	32 (55.17)	24 (64.86)	0.349
Female, n (%)	26 (44.83)	13 (35.14)	—
WHR	1.00 ± 0.10	0.97 ± 0.09	0.139
BMI (kg/m²)	28.33 ± 5.35	26.07 ± 5.36	0.048
Glucose and insulin-related markers			
Glucose (mmol/L)	4.57 (4.12–6.27)	3.86 (3.47–4.18)	<0.001
Insulin (µIU/mL)	18.18 (17.12–25.07)	17.97 (16.49–21.67)	0.401
HOMA-IR	4.45 (3.26–7.60)	3.09 (2.67–3.95)	<0.001
Lipid-related and atherogenic markers			
TC (mmol/L)	5.44 (4.85–6.58)	4.91 (4.56–5.45)	0.004
TG (mmol/L)	1.79 (1.59–2.15)	1.24 (1.15–1.36)	<0.001
LDL-C (mmol/L)	2.74 (2.32–3.37)	2.48 (2.26–2.77)	0.022
HDL-C (mmol/L)	1.01 (0.85–1.29)	1.01 (0.93–1.15)	0.973
NEFA (mmol/L)	0.92 (0.83–1.06)	0.86 (0.80–0.90)	0.002
AIP	0.25 (0.14–0.38)	0.09 (0.04–0.15)	<0.001

Footnotes: Data are presented as mean ± SD for normally distributed variables, median (IQR) for non-normally distributed variables, or n (%) for categorical variables. HOMA-IR was calculated as fasting insulin (µIU/mL) × fasting glucose (mmol/L) / 22.5. Comparisons between TyG groups were performed using Student's *t*-test for normally distributed continuous variables, the Mann–Whitney U test for non-normally distributed continuous variables, and the chi-square test for sex distribution. The *p* value for sex refers to the overall male/female distribution.

Abbreviations: AIP, atherogenic index of plasma; BMI, body mass index; HDL-C, high-density lipoprotein cholesterol; HOMA-IR, homeostasis model assessment of insulin resistance; IQR, interquartile range; LDL-C, low-density lipoprotein cholesterol; NEFA, non-esterified fatty acids; SD, standard deviation; TC, total cholesterol; TG, triglycerides; TyG, triglyceride–glucose index; WHR, waist-to-hip ratio.

Figure S1. Adjusted log-transformed fasting insulin across the continuous TyG index according to VDR rs7975232 genotype.

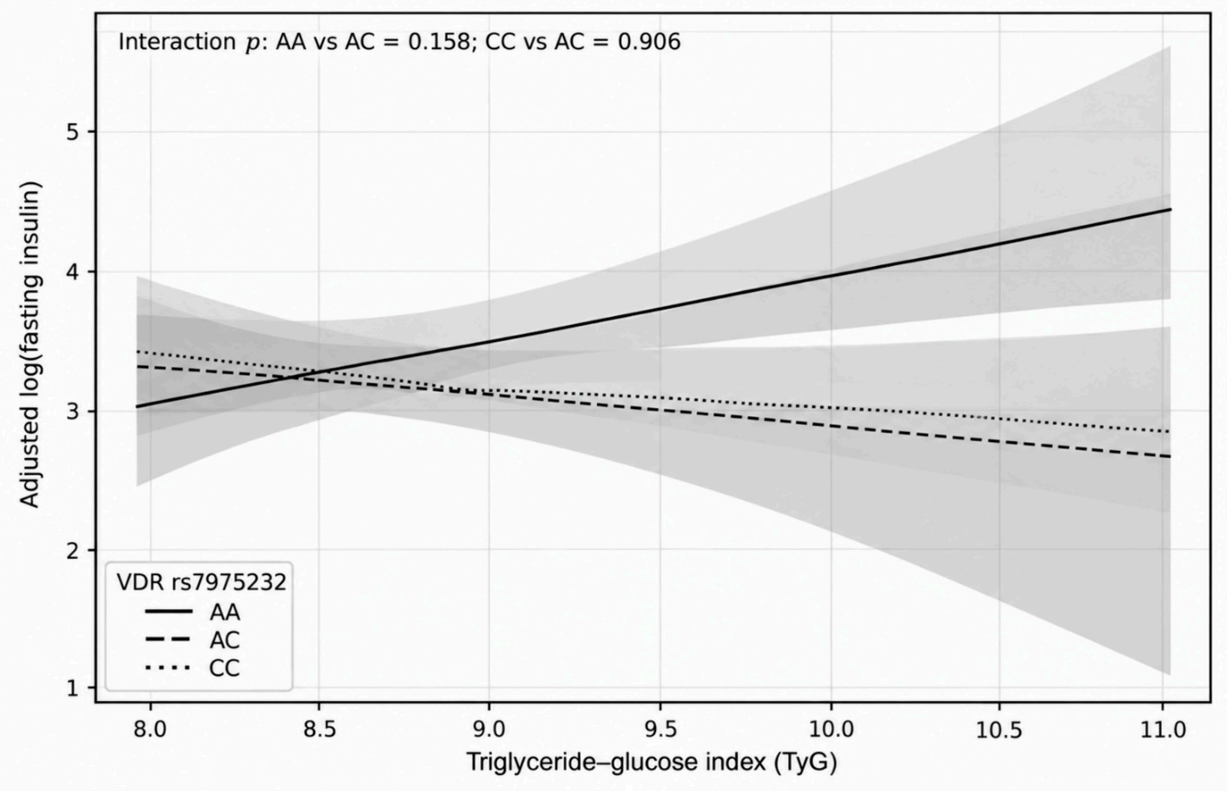

Footnotes: Predicted log-transformed fasting insulin values were obtained from a multivariable linear regression model adjusted for age, sex, and waist-to-hip ratio. Shaded areas represent 95% confidence intervals.

Abbreviations: TyG, triglyceride–glucose index; VDR, vitamin D receptor.

Figure S2. Adjusted fasting glucose concentrations across the continuous TyG index according to VDR rs7975232 genotype.

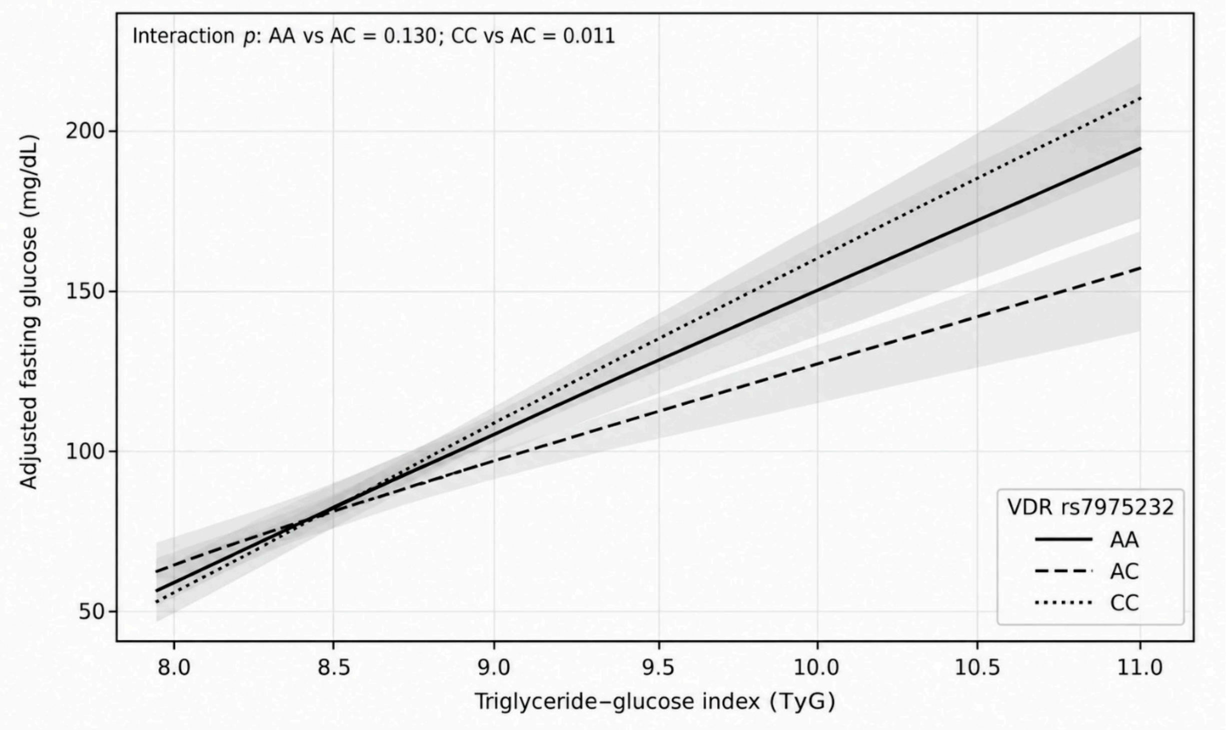

Footnotes: Predicted fasting glucose concentrations were obtained from a multivariable linear regression model adjusted for age, sex, and waist-to-hip ratio. Shaded areas represent 95% confidence intervals.

Abbreviations: TyG, triglyceride–glucose index; VDR, vitamin D receptor.
